# Binge-eating disorder in students: high prevalence and strong link to impulsive and compulsive traits

**DOI:** 10.1017/S1092852921000882

**Published:** 2021-10-18

**Authors:** Jeremy E. Solly, Samuel R. Chamberlain, Katherine Lust, Jon E. Grant

**Affiliations:** 1 Department of Psychiatry, University of Cambridge, UK; 2 Cambridge University Hospitals NHS Foundation Trust, Cambridge, UK; 3 Department of Psychiatry, Faculty of Medicine, University of Southampton; and Southern Health NHS Foundation Trust, Southampton, UK; 4 Boynton Health Service, University of Minnesota USA; 5 University of Chicago, Department of Psychiatry and Behavioral Neuroscience, Chicago, IL USA

## Abstract

**Objective:**

Binge Eating Disorder (BED) is the most common eating disorder, and is associated with significant comorbidity, with university students being particularly vulnerable. We aimed to assess associations of BED with a wide range of comorbidities and measures of impulsivity and compulsivity in university students, to gain better understanding of its prevalence, correlates and pathophysiology.

**Method:**

We carried out an internet-based survey, assessing presence of BED using a validated structured self-report diagnostic tool, demographics, substance use, impulsive behaviours, psychiatric history and measures of impulsivity and compulsivity. Approximately 10,000 students were invited to take part. Group differences between students with current BED and students without BED were investigated.

**Results:**

3415 students completed the survey, with 83 (2.4%) screening positive for BED. BED was associated with female gender, hazardous/harmful alcohol use, depression and anxiety symptoms, low self-esteem, post-traumatic stress disorder, attention-deficit/hyperactivity disorder, treatment for psychological/emotional problems (including prescribed medication) and trait impulsivity and compulsivity. However, the largest effect sizes were evident for associations with trait impulsivity and compulsivity.

**Conclusions:**

The associations of BED with trait impulsivity and compulsivity implicate these latent phenotypes in its pathophysiology. The identified links between BED and a wide range of mental disorders highlight the need to screen for disordered eating in student populations, including when students present with other mental health conditions.

## Introduction

Binge eating disorder (BED) is the most common typical eating disorder and is characterized by recurrent episodes of binge eating without purging, leading to marked distress.^1–4^ It is also associated with significant comorbidity; most individuals with lifetime BED meet criteria for other mental disorders and it is linked to physical comorbidities such as obesity and diabetes.^5–7^ The mean age of onset for BED is later than for anorexia nervosa and bulimia nervosa, occurring in young adulthood,^5,6^ and longitudinal data suggest that earlier detection and treatment might improve outcomes and reduce healthcare costs.^8^

Young adulthood is associated with the onset of a range of mental disorders, including BED, and university students may be particularly vulnerable, due to stressors such as academic pressure and major life changes.^9^ Improving understanding of BED rates and correlates in university students is therefore important in identifying risk factors and levels of need in this higher-risk group. Studies of student populations have reported rates of BED between 0 and 12.9%, depending on diagnostic criteria and the specific population studied,^10–18^ while prevalence of binge eating behaviours has been reported to be as high as 50% in female undergraduates.^19^ In student populations, binge eating has been associated with functional impairment,^20^ mental health problems and lower academic performance^21^ and so may have long-term consequences.

Impulsivity and compulsivity are latent constructs implicated in a number of mental disorders, as well as being highly relevant in normative populations.^22^ Impulsivity refers to a tendency to undertake behaviours/actions that are inappropriate, premature or risky,^23^ while compulsivity refers to repetitive acts that are performed according to rigid rules or in a habitual fashion.^24^ Both impulsivity and compulsivity are thought to be core features relevant to understanding of BED, as evidenced by findings from cognitive tasks, neuroimaging and trait impulsivity scales.^25–28^ Higher impulsivity and compulsivity may also be related to BED severity, as non-planning impulsivity has been found to correlate with binge eating frequency^29^, while higher levels of obsessiveness and compulsiveness of binge eating-related thoughts and behaviours, as well as more frequent binge eating, correlate with higher scores on the global clinical disease severity scale.^30^ It has not previously been possible to carry out trans-diagnostic measurement of trait compulsivity in normative and clinical populations due to lack of appropriate measures; however, reliable scales have recently been developed, allowing dimensional measurement of compulsivity in large sample sizes.^31^

Although a number of studies have assessed BED in university students either alone or with a small number of comorbidities, there are less data regarding associations of BED with a wider range of comorbidities in this population.^21^ In addition, although compulsivity is thought to be a core feature implicated in BED, it has not often been measured in such student studies. Therefore, this study aimed to examine the prevalence of BED in a large sample of university students, its demographic and clinical associations, and relationships to measures of trait impulsivity and compulsivity.

## Methods

The Internet-based survey was conducted at Boynton Health Services at the University of Minnesota, USA. Study procedures were conducted in accordance with the Declaration of Helsinki. The study was approved by the University of Minnesota’s Institutional Review Board.

### Participants

Email invitations were sent to 10,000 randomly selected students, from a total of approximately 60,000 students, at a large Midwestern university over three weeks in fall 2016. 9449 were successfully received. On responding to the email, students were informed via an online interface that participation was confidential, those completing the survey would be entered into a prize draw and 10 participants would be randomly selected to receive prizes (3 would receive tablet computers, 4 would receive $250 gift certificates, 2 would receive $500 gift certificates and 1 would receive a $1000 gift certificate). They were assured that contact details for the prize draw would be stored separately from their survey responses to ensure confidentiality. The survey was presented following provision of consent. Students were required to review, but not to answer, all questions to be eligible for the prize draw. 3659 students completed the survey. After one week, a reminder email was sent to selected students.

### Survey overview

The survey took approximately 30 minutes to complete. BED was assessed using the self-report Minnesota Impulse Control Disorders interview (MIDI) in which subjects first responded to a general question about the disorder. If they responded yes, further questions were asked based on diagnostic criteria and positive responses to all questions indicated presence of BED. Although, the MIDI criteria for BED have not been examined against other measures, the questions mirror the DSM-% criteria and thus it has good face validity. Furthermore, the MIDI shows high test-retest reliability, good concurrent validity against a gambling disorder interview and trait impulsivity scales and good divergent validity.^32^ Participants were classified into two groups: students meeting criteria for current BED and students without BED, based on the MIDI. As outlined in further detail below, the survey collected contextual information about demographics, substance use and impulsive behaviours/psychiatric history, and assessed measures of impulsivity and compulsivity.

### Demographics

Demographic information collected included gender, year in college (undergraduate or graduate), race/ethnicity and Grade Point Average (GPA) (<3.00 or ≥3.00).

### Substance use

Participants were asked about frequency of e-cigarette use and alcohol consumption, and age at first use of cigarettes/nicotine. They were asked if they had used any of the following and the frequency: non-prescription amphetamines, cocaine, opiates, inhalants, sedatives, marijuana, or prescription pain medication. Participants completed the Alcohol Use Disorders Identification Test (AUDIT) (with a score ≥8 indicating potentially hazardous or harmful alcohol use)^33^ and the Drug Abuse Screening Test (DAST-10) (with a score of 3 indicating a positive screen for a drug use disorder).^34,35^ They were also asked whether they had been treated for drug/alcohol use problems.

### Impulsive behaviours and psychiatric history

Participants were asked how many caffeinated soft drinks they had consumed over the past week; whether they had been treated for psychological/emotional problems; and whether they were currently taking prescribed mental health medication(s). Gambling disorder was assessed using the MIDI. Participants also completed the following previously validated questionnaires: Patient Health Questionnaire (PHQ-9) (with a score ≥10 indicating depressive symptoms of moderate severity or greater)^36^; Primary Care PTSD Screen (PC-PTSD) (with a score ≥3 indicating probable post-traumatic stress disorder, PTSD)^37^; Generalized Anxiety Disorder 7 (GAD-7) (with scores indicating: 0-4 no anxiety, 5-9 mild anxiety, 10-14 moderate anxiety and 15-21 severe anxiety)^38^; Adult ADHD Self-Report Scale (ASRS-v1.1) Part A (6 questions screening for attention-deficit/hyperactivity disorder, ADHD), where positive screen was based on previous definitions^39,40^; and the Rosenberg Self-Esteem Scale (RSES) (with a score <15 indicating low self-esteem).^41^

### Impulsivity and compulsivity

The Barratt Impulsiveness Scale, Version 11 (BIS-11) was used to assess impulsivity and provided three dimensional scores for impulsivity: attentional, motor and non-planning.^42,43^ The Cambridge-Chicago Compulsivity Trait Scale (CHI-T) assessed compulsivity and provided a total compulsivity score.^44^

### Statistical analysis

Respondents who answered the BED MIDI module (3415 out of 3659) were included in analyses. Likelihood ratio tests and analysis of variance (ANOVA) were used to explore associations of current BED with survey items. Effect sizes (measures of magnitude of the association between two variables) were reported using Cramer’s V (V) or Cohen’s D (D). V varies between 0 and 1, where values between .1-.2 suggest a weak association, between .2-.4 suggest a moderate association and above .4 suggest a relatively strong association.^45^ D of .2 is commonly interpreted as a small effect, .5 as a medium effect and .8 as a large effect.^46^ SPSS (version 24, IBM Corp) was used for all analyses. Statistical significance was defined as *P* <.05, with Bonferroni correction for the number of measures in each table. Missing data were missing completely at random (MCAR), and the sample was large. Therefore, the analysis was conducted using list-wise deletion where missing data occurred.

## Results

3415 college students were included. Of these, 83 (2.4%) had a positive screening outcome for BED, based on the relevant MIDI module. Current BED was associated with female gender. Other demographic factors (year in college, racial/ethnic group and GPA) did not differ significantly between those with versus without BED (Table 1).

BED was associated with higher AUDIT scores but not with frequency of alcohol consumption, treatment for drug/alcohol use problems or use of any other substances included in the survey (Table 2).

Students with current BED were more likely to have been treated for psychological/emotional problems and to be taking prescribed mental health medications. Questionnaires demonstrated that BED was associated with higher scores in the PHQ-9 and GAD-7, lower RSES scores, and higher likelihoods of PTSD and ADHD. BED was not associated with consumption of caffeinated soft drinks or with presence of gambling disorder (Table 3).

Students with current BED had higher BIS-11 total scores, including higher scores for each subscale. BED was also associated with higher CHI-T scores (Table 4).

## Discussion

This large study of university students assessed associations between BED and demographic and mental health measures. We found that 2.4% of students had a positive screen for current BED, which is in line with the lower end of estimates by previous studies in university student populations.^10–18^ BED was more likely in females, consistent with previous findings.^2^

The largest effect sizes in this study were seen when examining the associations of BED with trait impulsivity and compulsivity. Students with BED had significantly higher BIS-11 and CHI-T scores with medium-large effect sizes (D = .660 and .656, respectively), including higher scores in all three BIS-11 subscales. Deficits in tasks assessing inhibitory control and cognitive flexibility have previously been reported in BED, supporting involvement of impulsive/compulsive tendencies in its pathophysiology.^27^ In addition, neuroimaging findings have implicated brain regions such as the prefrontal cortex and striatum in BED, suggesting parallels with other impulsive/compulsive-related disorders such as substance use or behavioural addictions.^27,47–49^ Higher BIS-11 scores have previously been associated with BED.^50^ However, to our knowledge, this is the first study to investigate trait compulsivity in BED, as self-report measures designed to assess compulsivity across disorders have only recently been developed.^31^ Prior work has typically used scales unsuitable for measuring compulsivity, such as relying on measures of obsessive-compulsive symptoms. In future, these associations should be tested in a range of populations, using both self-report measures and cognitive tasks. It would also be valuable to conduct longitudinal research to examine whether these traits exist prior to BED and confer vulnerability to developing it.

Regarding substance use, BED was associated with higher AUDIT scores, consistent with previously reported links between BED and alcohol use disorder.^51^ However, the effect size was very small (V = .054) and there was no association between BED and frequency of alcohol consumption or treatment for drug/alcohol problems. As the AUDIT captures information regarding drinking behaviours as well as frequency,^33^ the higher AUDIT scores identified here may indicate a possible relationship between BED and binge drinking. Indeed, there is evidence of a direct relationship between binge eating and binge drinking in undergraduate students^52^ and this association could be mediated either via shared neurobiology or through use of these behaviours to avoid or delay unpleasant affective states.^51–53^ Given this possible relationship between binge eating and binge drinking, and the identification of higher AUDIT scores in students with BED in this study, it may be important to screen for other binge behaviours in individuals with BED.

BED was associated with a range of other mental disorders in this study. Students with BED reported higher levels of anxiety and depressive symptoms, lower self-esteem and higher likelihoods of PTSD and ADHD. They were also more likely to have been treated for psychological/emotional problems and to be taking prescribed mental health medications. However, and in contrast to the links with compulsivity and impulsivity at the trans-diagnostic level, all these associations had small effect sizes, with V between .076 and .151. These findings are in line with previous literature, which has reported that a majority of individuals with BED meet lifetime criteria for other mental disorders.^5^ Depressive symptoms and low self-esteem have been linked to self-criticism and the over-evaluation of shape and weight in BED,^54^ as well as to a greater likelihood of later reporting binge eating in a longitudinal study,^55^ suggesting that they might be related to the development of BED. Lifetime prevalence of BED is higher in individuals with PTSD compared to people with no trauma exposure and so binge eating may act as a mechanism to cope with PTSD symptoms or trauma.^56^ Prior literature also supports a relationship between binge eating and ADHD, although the nature of the association is unclear^57–59^ and may be accounted for by comorbid psychopathology.^60^ Taken together, the findings presented here support previous reports of comorbidity between BED and multiple other mental disorders. Students with BED in this study were more likely to have been treated for psychological/emotional problems than students without BED, in line with prior research in BED; however, evidence suggests that only a minority of individuals with BED receive treatment specifically for their eating disorder.^5,61^ This may be related to low levels of recognition, as one internet-based survey found that only a small minority (3.2%) of respondents from the general population meeting criteria for BED had received a formal diagnosis.^62^ Therefore, our findings highlight the need to screen for disordered eating in individuals presenting with a range of mental disorders, to facilitate access to specific support.

Major strengths of this study are its large sample size, the wide variety of comorbidities included, the use of a self-report BED tool adapted from a validated clinical interview (the MIDI), and the collection of a trans-diagnostic compulsivity measure (as well as other salient parameters). An important limitation is the anonymous, self-report design, which may be less accurate than face-to-face clinical assessment, including in terms of diagnosing BED accurately. However, this design may have facilitated participants disclosing information regarding their mental health and substance misuse more openly than they might have done in a face-to-face research study. In addition, as the survey was cross-sectional, causality cannot be shown. The response rate to the survey was 36.1% and it is not possible to confirm whether these findings generalize to the whole sample. As the survey was anonymous, it is not possible to compare the demographic information of those who completed the survey and those who did not. Those students who completed the survey, however, were similar on demographic variables to the larger university population.

To conclude, in a large sample of university students, we found a BED prevalence of 2.4% and BED was more likely in females. BED was associated with trait impulsivity and compulsivity with medium-large effect sizes, implicating these latent phenotypes in its pathophysiology. BED was also associated, with smaller effect sizes, with higher AUDIT scores, depressive and anxiety symptoms, PTSD, ADHD, low self-esteem and treatment for psychological/emotional problems (including prescribed medication). The identified associations with impulsivity and compulsivity strengthen current understanding of the neurobiological underpinnings of BED, while the associations with a wide range of mental disorders, albeit with small effect sizes, highlight the need to screen for disordered eating in a range of psychiatric presentations.

## Figures and Tables

**Table 1:** Demographics of University Students based on Binge Eating Disorder

Variable	Students with current Binge Eating Disorder (n=83)	Students without Binge Eating Disorder (n=3332)	Likelihood Ratio	*P* value	Effect Size Cramer’s V
Sex					
Female	64 (77.1)	1975 (59.3)	LR=17.374	.001*	.067
			df=3		
Year in college					
Undergraduate	56 (67.5)	2200 (66.0)	LR=.979	.613	
Graduate	27 (32.5)	1113 (33.4)	df=2		
Race/ethnicity					
Caucasian	62 (74.7)	2481 (75.8)	LR=.051	.821	
			df=1		
Grade Point Average, GPA					
Less than 3.00	11 (13.3)	336 (10.2)	LR=.755	.385	
3.00 or higher	72 (86.7)	2956 (89.8)	df=1		

All numbers are N (%) unless otherwise stated.

* *P* value significant with Bonferroni correction; critical *P* = .05/4 = .125.

**Table 2:** Alcohol, Tobacco and Drug Use Based on Binge Eating Disorder

Variable	Students with current Binge Eating Disorder (n=83)	Students without Binge Eating Disorder (n=3332)	Likelihood Ratio	*P* value	Effect Size Cramer’s V
Age at first use of cigarettes or nicotine					
Never used	49 (59.0)	1996 (59.9)	LR=3.757	.289	
Less than 14 years	9 (10.8)	184 (5.5)	df=3		
15-17 years	12 (14.5)	527 (15.8)			
18 years or older	13 (15.7)	625(18.8)			
Frequency of e-cigarette use					
Never	19 (55.9)	726 (54.4)	LR=3.208	.524	
Not within past year	9 (26.5)	287 (21.5)	df=4		
Rarely	3 (8.8)	227 (17.0)			
Occasionally	1 (2.9)	60 (4.5)			
Daily	2 (5.9)	34 (2.5)			
Frequency of alcohol consumption					
Never	13 (15.7)	620 (18.6)	LR=3.731	.444	
Monthly or less	21 (25.3)	629 (18.9)	df=4		
2-4 times a month	23 (27.7)	1074(32.2)			
2-3 times a week	22 (26.5)	765 (23.0)			
4+ times a week	4 (4.8)	243 (7.3)			
Non-prescription amphetamines					
Never	76 (91.6)	3249 (97.9)	LR=12.930	.012	
In past, not within past 12 months	3 (3.6)	40 (1.2)	df=4		
Rarely	2 (2.4)	20 (0.6)			
Occasionally	2 (2.4)	4 (0.1)			
Daily	0 (0.0)	5 (0.2)			
Cocaine					
Never	74 (89.2)	3051 (92.3)	LR=2.825	.419	
In past, not within past 12 months	5 (6.0)	153 (4.6)	df=3		
Rarely	4 (4.8)	82 (2.5)			
Occasionally	0 (0.0)	21 (0.6)			
Daily	0 (0.0)	0 (0.0)			
Opiates					
Never	82 (98.8)	3268 (98.6)	LR=.717	.949	
In past, not within past 12 months	1 (1.2)	34 (1.0)	df=4		
Rarely	0 (0.0)	6 (0.2)			
Occasionally	0 (0.0)	3 (0.1)			
Daily	0 (0.0)	5 (0.2)			
Inhalants					
Never	80 (96.4)	3264 (98.6)	LR=5.253	.154	
In past, not within past 12 months	2 (2.4)	34 (1.0)	df=3		
Rarely	0 (0.0)	6 (0.2)			
Occasionally	1 (1.2)	3 (0.1)			
Daily	0 (0.0)	5 (0.2)			
Sedatives					
Never	74 (89.2)	3163 (95.4)	LR=6.326	.176	
In past, not within past 12 months	5 (6.0)	85 (2.6)	df=4		
Rarely	3 (3.6)	38 (1.1)			
Occasionally	1 (1.2)	23 (0.7)			
Daily	0 (0.0)	7 (0.2)			
Marijuana					
Never	38 (45.8)	2034 (61.1)	LR=9.868	.043	
In past, not within past 12 months	11 (13.3)	359 (10.8)	df=4		
Rarely	20 (24.1)	442 (13.3)			
Occasionally	11 (13.3)	369 (11.1)			
Daily	3 (3.6)	123 (3.7)			
Prescription pain medication					
Never	75 (90.4)	3069 (92.6)	LR=2.412	.660	
In past, not within past 12 months	5 (6.0)	177 (5.3)	df=4		
Rarely	3 (3.6)	52 (1.6)			
Occasionally	0 (0.0)	11 (0.3)			
Daily	0 (0.0)	4 (0.1)			
AUDIT score ≥8 (%)	33 (39.8)	819 (24.6)	LR=8.959	.003*	.054
			df=1		
DAST-10 score ≥3 (%)	10 (12.2)	273 (8.2)	LR=1.489	.222	
			df=1		
Has been treated for drug/alcohol use problems					
Yes	4 (4.8)	59 (1.8)	LR=2.922	.087	
			df=1		

All numbers are N (%) unless otherwise stated.

* *P* value significant with Bonferroni correction; critical *P* = .05/13 = .004.

AUDIT = Alcohol Use Disorders Identification Test (AUDIT); DAST-10 = Drug Abuse Screening Test.

**Table 3:** Impulsive Behaviours and Psychiatric History Based on Binge Eating Disorder

Variable	Students with current Binge Eating Disorder (n=83)	Students without Binge Eating Disorder (n=3332)	Likelihood Ratio	*P* value	Effect Size Cramer’s V
Amount of caffeinated soft drinks consumed over the past week					
Never	38 (45.8)	1605 (48.2)	LR=2.910	.714	
1-2 drinks	27 (32.5)	1070 (32.1)	df=5		
3-6 drinks	9 (10.8)	430 (12.9)			
7-12 drinks	5 (6.0)	149 (4.5)			
13-23 drinks	2 (2.4)	53 (1.6)			
24 or more drinks	2(2.4)	25(0.8)			
Gambling disorder?					
Positive screen	0 (0.0)	14 (0.4)	LR=.691	.406	
			df=1		
Has been treated for psychological/emotional problems					
Yes	49 (59.0)	968 (29.1)	LR=31.228	<.001*	.101
			df=1		
Currently taking prescribed mental health medication(s)					
Yes	29 (34.9)	441 (13.2)	LR=24.381	<.001*	.097
			df=1		
PHQ-9 total					
Score ≥10	12 (14.5)	140 (4.3)	LR=12.799	<.001*	.076
			df=1		
PTSD					
Positive screen	30 (36.6)	457 (13.8)	LR=25.851	<.001*	.100
			df=1		
GAD-7 total					
No Anxiety (score 0-4)	26 (32.1)	1927 (58.8)	LR=24.368	<.001*	.087
Mild (score 5-9)	29 (35.8)	782 (23.9)	df=3		
Moderate (score 10-14)	14 (17.3)	358 (10.9)			
Severe (score 15-21)	12 (14.8)	210 (6.4)			
ADHD					
Positive screen	31 (37.3)	560 (17.0)	LR=18.953	<.001*	.083
			df=1		
RSES total					
Score ≥15	41 (51.2)	2797 (86.1)	LR=53.479	<.001*	.151
			df=1		

All numbers are N (%) unless otherwise stated.

* *P* value significant with Bonferroni correction; critical *P* = .05/9 = .006.

ADHD = Attention-Deficit/Hyperactivity Disorder; GAD-7 = Generalized Anxiety Disorder 7; PHQ-9 = Patient Health Questionnaire; PTSD = Post-Traumatic Stress Disorder; RSES = Rosenberg Self-Esteem Scale.

**Table 4:** Impulsivity and Compulsivity Based on Binge Eating Disorder

Variable	Students with current Binge Eating Disorder (n=83)	Students without Binge Eating Disorder (n=3332)	ANOVA	*P* value	Effect Size Cohen’s D
CHI-T total	17.92 (14.9)	9.09 (13.4)	F(1,3384)=34.49	<.001*	.656
BIS-11 total	65.98 (11.2)	59.27 (10.1)	F(1,3195)=34.486	<.001*	.660
Attentional impulsiveness	18.84 (4.5)	16.14 (3.9)	F(1,3288)=37.706	<.001*	.683
Non-planning impulsiveness	25.51 (4.6)	22.87 (4.7)	F(1,3282)=24.855	<.001*	.558
Motor impulsiveness	21.77 (4.3)	20.29 (3.9)	F(1,3296)=11.113	.001*	.378

Data refer to mean and (standard deviation).

* *P* value significant with Bonferroni correction; critical *P* = .05/5 = .01.

BIS-11 = Barratt Impulsiveness Scale, Version 11; CHI-T = Cambridge-Chicago Compulsivity Trait Scale.
